# A Split-Mouth Design Comparison of Guided and Conventional Endodontic Treatments in Calcified Lower Incisors: An In Vivo Pilot Study

**DOI:** 10.7759/cureus.103011

**Published:** 2026-02-05

**Authors:** Rita J Ghaleb, Nabil Ghosn, Roula El Hachem, Carla Zogheib, Alfred Naaman, Issam Khalil, Kenneth Jordy, Marc Krikor Kaloustian

**Affiliations:** 1 Endodontics, Saint Joseph University, Beirut, LBN; 2 Craniofacial Research, Saint Joseph University, Beirut, LBN; 3 Endodontics, University of Siena, Siena, ITA

**Keywords:** guided endodontics, lower incisor, pulp canal calcification, substance loss, surgical guide

## Abstract

Aim: This in vivo pilot study aimed to compare the conventional endodontic technique (CET) to the guided endodontic technique (GET) in lower calcified incisors by focusing on the detection of root canals, the amount of dental substance loss, and treatment duration.

Methods: Seven patients with two calcified lower incisors with a single canal requiring root canal treatment were selected. For each patient, one incisor underwent CET, and the contralateral incisor underwent GET using a customized sleeve. The intracanal space was volumetrically measured using the indirect post-core technique for comparison. The duration was recorded from the start of the treatment until the root canal orifice was detected. Statistical analysis was conducted using IBM SPSS Statistics (version 25.0; IBM Corp., Armonk, NY, USA), employing the Wilcoxon signed-rank test (p < 0.05).

Results: For the 14 treated teeth, the calcification level was presented at the third middle part of the root canal. All 14 teeth had negotiable canals except one, which was treated using the CET. The CET presented a significantly higher intracanal volume (7.8 ± 3.3 mm^3^ vs. 5.3 ± 1.3 mm^3^, p = 0.018) and treatment duration compared to the GET (69.0 ± 57.4 minutes vs. 14.1 ± 8.2 minutes; p = 0.018).

Conclusion: GET offers a more predictable and efficient treatment for calcified lower incisors than CET, with significantly less substance loss and shorter treatment duration.

## Introduction

Dental pulp is a vital tissue that can be prone to physiological or pathological changes. Denticles, pulp stones, and other calcifications are frequently observed within "healthy" pulps. The American Association of Endodontists categorizes teeth with “pulpal obliteration” as having a moderate to high difficulty level of treatment, and their failure rate ranges from 10% to 19% [1,2]. Pulpal calcification alone is not a reason for endodontic treatment. Root canal treatment (RCT) is recommended and deemed necessary only in the presence of a peri-radicular pathology or if the affected tooth shows symptoms. To manage these complex cases, guided endodontics presents a potential solution. This approach involves integrating Digital Imaging and Communications in Medicine (DICOM) files from cone-beam computed tomography (CBCT) scans with stereolithography (STL) files from digital surface scans using specialized planning software [3]. This software facilitates the design of a custom guide incorporating a metal sleeve, ensuring precise bur guidance during the procedure [4]. With several in vitro studies [5-10] and case reports [11-14] published, this approach is becoming more prevalent in the literature. However, all these studies used a non-customized sleeve, which exhibited some intrinsic errors, such as deviation, and employed drills with a large tip diameter, resulting in over-enlargement and ledge formation. In addition, there is no information regarding the detection and negotiation of root canals, dentinal substance loss, and treatment duration using the guided endodontic technique (GET) compared with the conventional endodontic technique (CET) for RCTs in clinical practice [15]. The objective of this in vivo pilot study was to compare CET and GET, employing a small-tip drill with a customized sleeve, in lower calcified incisors, focusing on canal detection and negotiation, dentinal substance loss, and treatment duration.

## Materials and methods

The research was conducted in compliance with the Helsinki Declaration of 1975, as revised in 2000, and the study protocol was approved by the Ethics Committee of Saint Joseph University (USJ-2023-213). The study was registered in the database at www.clinicaltrials.gov (National Clinical Trial (NCT) number 06484218).

To determine the sample size, a power analysis was performed using G*Power software (Heinrich-Heine-Universität Düsseldorf, Düsseldorf, Germany) to perform a Wilcoxon signed-rank test (matched pairs), considering a power of 80%, an alpha error of 5%, and an effect size of 0.80. The minimum sample size required was 12 teeth.

Patients with two calcified lower incisors (contralateral incisors) requiring RCT were recruited at the Endodontic Department. Patients were informed about the study, including detailed treatment procedures and potential outcomes, and they were asked to sign an informed consent form.

Fourteen teeth were selected for inclusion in this study based on specific clinical and radiographic criteria. Only single-rooted mandibular incisors with a Vertucci type I canal configuration were included. Eligible teeth exhibited pulp canal obliteration extending from the cementoenamel junction (CEJ) for more than 5 mm into the radicular pulp canal space, as confirmed radiographically. All included teeth required RCT due to the presence of clinical symptoms, a periradicular lesion, or an endodontic-periodontic lesion. Additionally, only teeth with straight root canals, defined as a curvature of less than 5 degrees, and with no clinical mobility were selected.

Teeth were excluded if they presented with anatomical variations such as two roots or two canals (Vertucci type II or higher), as detected on preoperative CBCT. Teeth previously restored with full-coverage crowns or those that were severely carious were also excluded from the study.

Preoperative CBCT (Orthophos SL 3D, Dentsply, Charlotte, NC, USA) assessment of the concerned teeth was performed, and the coronal and sagittal slices were examined using the 3D Slicer software (Harvard, USA) to measure the calcification level. Based on these measurements, the calcification level was classified in relation to the length of the tooth as coronal (extends from the CEJ level down to the end of the first third of the root), middle (extends from the end of the first third level to the end of the second third of the root length), or apical (extends from the end of the second middle down to the apex). Each third of the canal (coronal, middle, and apical) was further subdivided into three equal segments, and the calcification level within each third was classified as first, second, or third according to its position along the length of that third.

Virtual planning of guided endodontic treatment

**Figure 1:**
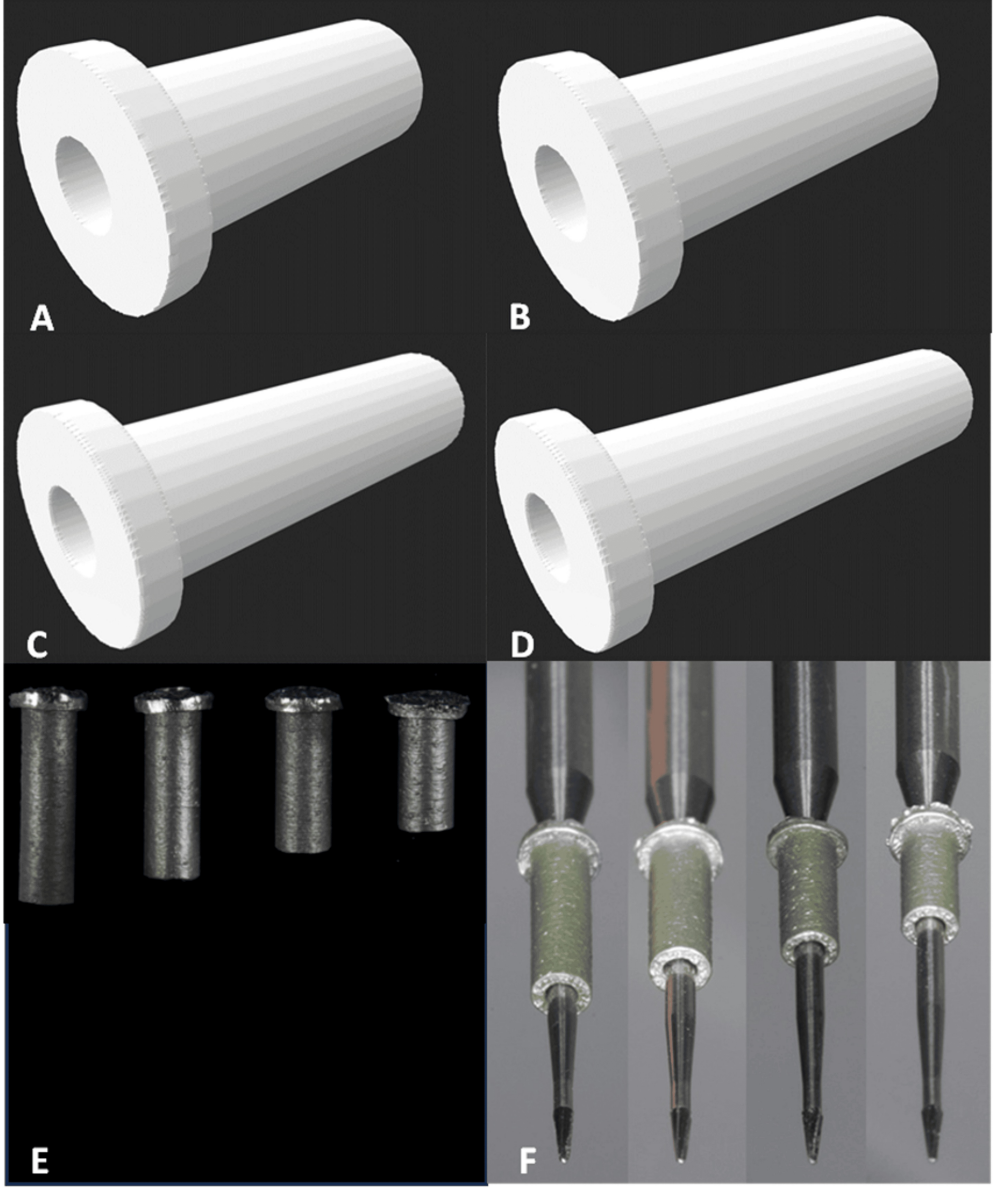
Sleeve design with a length of (A) 8 mm, (B) 7 mm, (C) 6 mm, and (D) 5 mm. (E) Laser-manufactured metal sleeves. (F) Adaptation between the drill and the sleeves with different lengths from 8 down to 5 mm.

The EG6 from SSWhite endo guide drills (SSWhite Dental, Lakewood, NJ, USA) was selected for drilling, characterized with a total length of 34 mm, a working length of 17 mm, a head length of 2.5 mm, and a tip diameter of 0.28 mm. A sleeve design specific to drill EG6 was initiated to maintain the axis during dentin trepanation. The sleeves were designed with an inner diameter of 1.1 mm, an external diameter of 2 mm, and different lengths (5, 6, 7, and 8 mm). The longest sleeve that allowed the drill to reach the level of calcification was selected whenever possible, as longer sleeves provide greater stability and more accurate guidance of the drill toward the target area within the root canal system. In cases of deeper calcification, a shorter sleeve was required to allow the drill to reach sufficient depth, as illustrated in Figure 1.

In all cases, a digital protocol was followed according to the method reported by Zehnder et al. [3] and Connert et al. [16]. A digital intraoral scan of the mandible was taken (3shape, Trios, Copenhagen, Denmark) to obtain a 3D virtual model. The 3D virtual model and CBCT images were then converted to a 3D STL file, imported into BlueSkyPlan (Libertyville, IL, USA), and matched using the alignment tool. The software was configured to create a customized virtual implant matching the dimensions of the EG6 drill. It was placed in the axis of the canal and in the middle of the tooth to provide a straight path to the canal. A virtual sleeve for guidance was placed, having the same measurements and design as that prepared for the drill EG6. Once the drill sleeve's location was established, a virtual template of the guide was generated. The virtual model was exported as an STL file to a 3D printer (Formlabs, Somerville, MA, USA) to be fabricated.

Sample preparation

One endodontist was tasked with preparing the CET and GET samples. For CET, a rubber dam was placed on several teeth, and the conventional access cavity was prepared under the microscope at 10x magnification (Leica M320, Wetzlar, Germany) through the incisal edge, as this approach facilitates straight-line access. A high-speed contra-angle handpiece associated with a long surgical bur 010 (Meisinger, Centennial, CO, USA) was used until the dentin was exposed. An X-ray was then taken to determine the burr penetration axis, which should be in the center of the root. After confirming the axis of penetration and exposing the dentin, the ultrasonic tip ED5 (Woodpecker-DTE, Guilin, China) was marked with a stopper based on the calcification level observed in the preoperative CBCT. It was then used gently up to the stopper level at 25x magnification, following the color variation between the dentin and the calcified tissues; the dentin appeared more yellow/gray, while the calcified tissues were whiter and more opaque [17]. This technique is known as the "road-mapping" technique. If the ultrasonic tip was used at the stopper level, but the canal was not successfully found, the patient was referred for limited field of view (FOV)-CBCT of the tooth area (Orthophos SL 3D). The ultrasonic tip was then redirected to the center of the root at the position indicated by the CBCT until the canal was found. When the root canal was accessed and negotiated, a periapical radiograph was obtained using a 08-K file (Dentsply Sirona, Ballaigues, Switzerland).

**Figure 2:**
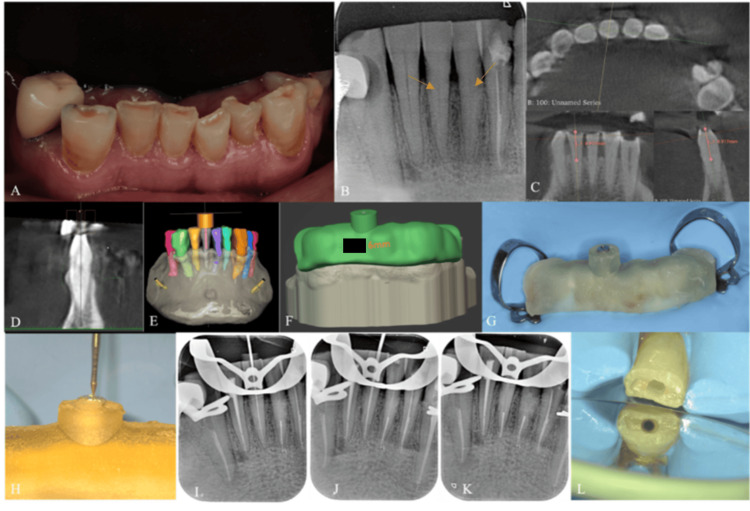
(A) Intraoral photo showing tissue loss due to severe bruxism on the anterior teeth. (B) Initial preoperative X-ray showing calcified root canals on teeth 31 and 41. (C) Measurement of calcification level on 3D slicer. (D) Advanced semi-automatic segmentation of tooth 41 and customized virtual implant presenting the drill’s position. (E) Virtual drill-sleeve’s location. (F) Virtual template of the guide with the patient’s initials and the length of the sleeve to be used engraved on it. (G) Endodontic guide placed after ensuring perfect isolation using a rubber dam. (H) Drilling through the endodontic guide and sleeve, drill of 0.28 mm tip diameter (SSWhite Dental). (I) Working length determination. (J) Cone fit. (K) Post-obturation of the apical 5 mm. (L) Guided access cavity preparation.

For the GET, once the stability of the guide was established, it was removed to give local anesthesia. To prevent any interference between the guide and the clamp, a rubber dam was positioned on several teeth to segregate the operating field. A small mark was placed on the guide on the tooth surface using an EG6 drill. The guide was removed, and a small cavity was drilled 3 mm into the enamel using a long surgical bur 010 (Meisinger). The guide was then replaced, and the access cavity was drilled using an EG6 drill mounted on a low-speed contra-angle handpiece at 30,000 rpm. Every 3 mm of progression, the cavity was rinsed, and the head of the bur was cleaned until it reached the canal. Irrigation was performed to avoid overheating of the dentin and accumulation of debris (Figure 2).

After reaching the canal in the CET and GET groups, a glide path was achieved with the NiTi Endostar Easy Path (14/04) (Poldent, Warsaw, Poland), and instrumentation of the canals was performed using the NiTi Endostar E3 Azure (20/04) instrument (Poldent), followed by the NiTi Endostar E3 Azure (25/04) instrument (Poldent). To ensure comparability between samples and decrease the risk of biased results due to increased tapered preparations, a final file 25/04 was used to shape all canals.

During treatment, the root canal was irrigated with 12 mL of 5.25% sodium hypochlorite (NaOCl). The final irrigation protocol involved 17% ethylenediaminetetraacetic acid (EDTA), activated for one minute within the canal using the EndoActivator (Dentsply Sirona) with a 25/04 tip. Saline was used as an intermediate rinse between the irrigants and as a final rinse at the end of the procedure.

The root canal was then dried using sterile paper points, and only the apical 5 mm was obturated using the warm vertical condensation technique with fine gutta-percha and cement (Sealite™ Regular, Acteon Group, Paris, France) using the 40/0.4 tip from the fast pack Eighteeth system (Eighteeth, Changzhou Sifary Medical Technology, Changzhou, China). The access cavity was then filled with a sterile Teflon tape and temporarily sealed with 4 mm Cavit G (3M ESPE, Seefeld, Germany).

In both techniques, the time was recorded from the beginning of the treatment, after placing the rubber dam and initiating the access cavity preparation, until the root canal was accessed and negotiated with a 08-K file or until the operator ended the (unsuccessful) treatment with the video recording. The planning time was excluded from the treatment duration in the GET samples.

Post-operative assessment

**Figure 3:**
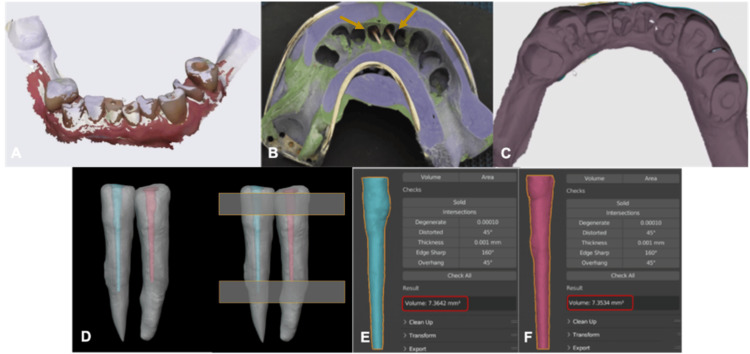
(A) Digital intraoral scan of the mandible presenting the access cavities of the treated teeth. (B) Heavy and light impression presenting the two posts and cores. (C) Digital scan of the impression. (D) The treated teeth presenting the intracanal impressions exported to the Blenderfordental software, reoriented, and aligned at the same level. (E) The intracanal impressions cut at the same level coronally and apically. (F) The software Blenderfordental showing the intracanal volume of teeth 41 and 31 defined by 7.3534 and 7.3642 mm³, respectively.

A digital intraoral scan of the mandible was performed (3shape, Trios), presenting the access cavities of the treated teeth. An impression of the intracanal space was obtained using the indirect post-and-core technique. The heavy and light impressions of the two posts and cores of the treated teeth were recorded. A digital scan of the impressions was performed. The last one was merged with the intraoral scan presenting the access cavities and with the preoperative CBCT using the BlueSkyPlan software to determine the anatomy of the treated teeth with the intracanal impression. The teeth were exported to Blenderfordental software (version 3.3 lts), reoriented, and aligned at the same level because some teeth were intruded or extruded. The intracanal impressions were cut at the same level to have the same length to avoid any volume inaccuracy due to the length difference. The volume of the intracanal space was measured using both techniques and the same Blenderfordental software. This volume represents substance loss (Figure 3).

Statistical analysis

Statistical analysis was performed using the IBM SPSS Statistics software version 25.0 (IBM Corp., Armonk, NY, USA). A descriptive analysis was carried out in which quantitative variables were presented using ranges (minimum-maximum), means, and standard deviations (SDs). Qualitative variables were expressed as frequencies and percentages. The Wilcoxon signed-rank test was used to identify potential significant differences between CET and GET in terms of volume and treatment duration. Statistical significance was set at p < 0.05.

## Results

In all 14 treated teeth, calcification was observed in the middle third of the root canal: six had the calcification at the first third (42.8%), four at the second third (28.6%), and four at the third level (28.6%). Notably, for all participants, both treated teeth presented the same level of calcification. For this reason, no specific consideration was given when deciding which tooth would be treated with GET or CET (Table 1).

**Table 1:** The results for the CET and GET regarding the detection of root canals, the substance loss (mm³), and treatment duration (TD) (mins) for all samples. Samples 1, 2, and 3 correspond to the teeth having a calcification level at the first third of the third middle part of the root canal, 4 and 5 to the second third, and 6 and 7 for the third level of the middle part of the root canal. CET: conventional endodontic technique; GET: guided endodontic technique

Sample	Detected canals	CET volume (mm³)	GET volume (mm³)	Volume difference (mm³)	Volume difference (%)	CET TD (mins)	GET TD (mins)	TD difference (mins)	TD difference (%)
1	Yes	7.3642	7.3534	0.0108	0.15	23	10	13	78.79
2	Yes	6.4457	6.2182	0.2275	3.59	20	8	12	85.71
3	Yes	4.081	3.8686	0.2124	5.34	25	4	11	144.83
4	Yes	5.733	3.9221	1.8109	37.51	70	12	58	141.46
5	Yes	6.5139	4.4409	2.073	37.85	80	15	65	136.84
6	Yes	10.4793	5.28	5.1993	65.98	100	23	77	125.2
7	No (CET), yes (GET)	13.7421	6.3016	7.4405	74.24	175	27	148	146.53

**Figure 4:**
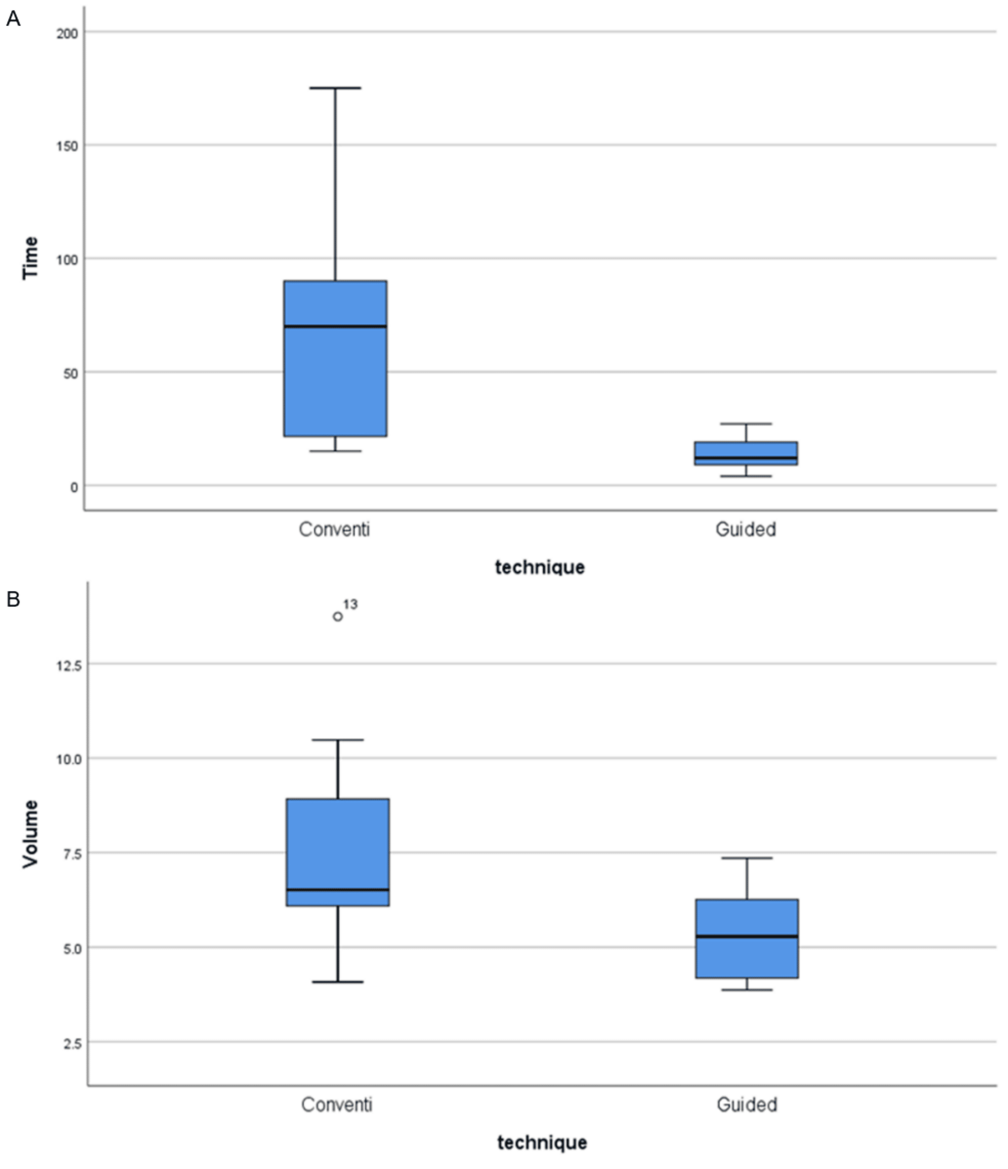
(A) TD for the different techniques (n = 14). (B) Volume for the different techniques (n = 14). TD: treatment duration

Seven teeth were treated using CET (n = 7) and another seven using GET (n = 7). All teeth had negotiable canals except for one that was treated using CET with the calcification level at the third level of the middle part. This unsuccessful case was treated by an endodontic apical surgery. Regarding substance loss, CET resulted in significantly higher intracanal volume (7.8 ± 3.3 mm³ vs. 5.3 ± 1.3 mm³, p = 0.018) and longer treatment duration (69.0 ± 57.4 minutes vs. 14.1 ± 8.2 minutes; p = 0.018) compared to GET. The findings are shown in Figure 4 and Table 2.

**Table 2:** TD for the different techniques (n = 14). SD: standard deviation

	Conventional endodontic technique (CET)		Guided endodontic technique (GET)		p-value
	Min-Max	Mean ± SD	Min-Max	Mean ± SD	
Treatment duration (TD)	15.0-175.0	69.0 ± 57.4	4.0-27.0	14.1 ± 8.2	0.018
Volume (mm³)	4.1-13.7	7.8 ± 3.3	3.9-7.4	5.3 ± 1.3	0.018

## Discussion

The accuracy of GET has been evaluated using various methods, including dentinal loss [5,6,8,18], measurements of linear and angular deviation [7,16], and drilling precision [19]. Previous in vitro studies have been conducted on 3D printed teeth or resin teeth. This pilot study is the first in vivo study to compare CET with GET in the treatment of lower calcified incisors by evaluating the detection and negotiation of root canals, the amount of dentinal substance loss, and treatment duration for each technique.

In the present in vivo study, one tooth type and a split-mouth design were considered to reduce the anatomical and intracanal volume variability between the samples [20], in contrast to other studies that used different tooth types and resin teeth [18]. Natural teeth provide a more realistic representation of human dental anatomy, including dentin characteristics, than resin teeth. The sensation of hardness between resin teeth and human teeth during drilling is very different, in addition to the shade of color variation between the dentin and calcified tissues.

This study utilized EG6 endo guide drills (SSWhite Dental), characterized by a 0.28 mm tip diameter, which minimized ledge formation in GET samples compared to other drills mentioned in the literature [4-7,11]. As a result, no ledges were observed in all the GET samples.

A 3D-printed customized metal sleeve was used, as opposed to all the previous case reports and in vitro studies [9,16,21,22] that used non-customized metal sleeves or where the authors were not clear concerning the dimensions of the sleeves utilized. A non-customized sleeve showed some intrinsic errors, such as the deviation defined by the angulation between the virtual burr axis and the actual burr axis, which is related to two main factors: the length of the sleeve and the gap between the drill and the sleeve. A longer sleeve and a smaller gap between the drill and sleeve reduce the intrinsic error [9,23]. In the present study, the longest sleeve that could reach the calcification level was selected for the GET. While varying sleeve lengths between cases could be seen as a limitation in terms of sample standardization, in this study, it may be considered a strength, given the demonstrated benefits of a longer sleeve. Moreover, the gap between the drill and sleeve was eliminated by customizing the sleeve based exactly on the drill measurement.

Dentinal loss was evaluated based on intracanal volume, measured after shaping with a 25/04 file and obturation of the apical 5 mm. To adhere to the “as low as reasonably achievable” (ALARA) principle, the indirect post-and-core technique was used. In contrast, other studies [5,6,8,18] measured intracanal volume immediately after negotiating the canal with a 08-K file, without shaping, using post-operative CBCT. However, the indirect post-and-core technique may not fully capture undercuts, recessed areas, or grooves created during drilling, potentially leading to an underestimation of volume. This limitation should be acknowledged, yet the technique remains widely used when post-operative CBCT and micro-computed tomography are not feasible in clinical studies. In this study, the shaping protocol influenced intracanal volume, but this step was necessary to reflect real clinical scenarios, as RCT inherently requires shaping.

The results of this study showed that six out of seven teeth (85.7%) were accessed and negotiated using the CET, while one tooth with a third-level calcification in the middle part of the canal remained untreated. Seven out of seven teeth (100%) were negotiated using GET.

These results are consistent with the high accuracy and success rates of GET reported in several previous studies [3,18]. The success rate of CET was greater than that recorded by Connert et al. [18], Krastl et al. [22], Vasudevan et al. [10], and Hildebrand et al. [6], which may be attributed to the fact that the present sample included human teeth, while other studies used 3D-printed teeth. The operator was able to access and negotiate the root canal using the “road-mapping” method, which consists of examining the color variation between dentin and calcified tissues under an operating microscope. Similar to other in vitro studies [5,6,8,18], the intracanal volume and treatment duration of untreated teeth from the CET were not excluded from the results. In the present study, preoperative CBCT showed that the canal was explored with the ultrasonic tip until the third level of the middle part. Since the target zone area for the intracanal volume measurement was above the apical 5 mm, which is similar to other successful samples, this particular sample was included in the results.

Moreover, the overall mean dentinal loss was significantly lower for the GET compared to the CET, consistent with previous in vitro studies [5,18], with a mean of 7.8 mm^3^ for the CET compared to 5.3 mm^3^ for the GET. However, the dentinal loss quantified in this study was marginally lower than that reported previously. Differences in tooth types, drill tip diameters, and volume measurement methods may be possible reasons for this variation. The sample was limited to the lower incisors, in contrast to other studies where the upper central incisors and canines were included. Lower incisors are generally smaller in size, with a narrower and shorter root canal than the root canals of other teeth. A smaller drill of 0.28 mm^3^ was used, as opposed to the larger drill of 1.3 mm^3^ [5,18]. In addition, the intracanal volume was measured using the indirect post-and-core technique, which may not capture the undercuts that refer to a recessed area or groove that was created during drilling, leading to an underestimation of the volume. This volume measurement technique was one of the limitations of this study.

The present study revealed that the CET resulted in a significantly longer treatment duration, with a mean of 69 minutes compared to the GET, with a mean of 14.1 minutes. Other studies have shown that the time required for an endodontic specialist was 21.8 minutes for the CET and 11.3 minutes for the GET [18]. The difference between these results was more prominent with CET than with GET. This difference may be attributed to the fact that the study was conducted in clinical conditions where treating real patients involves a higher level of responsibility, complexity, and variability, which naturally requires more time and effort.

Due to the small sample size, it was not possible to perform a statistical analysis of the intracanal volume and treatment duration in relation to the calcification level; however, some descriptive observations were made, and the results revealed that the GET outperformed the CET in terms of dentinal loss and treatment duration, with some results showing substantial differences, while others showing minimal differences depending on the calcification level in the first, second, and third levels of the middle part of the root canal, as shown in Table 1. In terms of dentinal loss, the difference between the two techniques was relatively small in the first third of the middle portion, ranging from 0.15% and 5.34%. However, the difference increased to an average of 37% in the second third of the middle section. Finally, the difference peaked at the third level of the middle part, ranging between 66% and 74%. In terms of treatment duration, the difference between the two techniques was much larger overall throughout all three aforementioned middle parts, as it ranged between 79% and 146%. However, it must be noted that the GET duration did not include the time spent designing and manufacturing the guide during the planning phase. None of the related studies examined the relationship between calcification level and the type of treatment. Larger sample sizes are necessary to perform a statistical analysis to better inform the decision-making process regarding the calcification level and the type of treatment to be chosen accordingly.

In the present study, it is paramount to explain the effect of the shaping protocol, EG6 drill, and ED5 insert on the results; the final shaping instrument used was characterized by a tip diameter of 0.25 mm and a taper of 4%, which means that at D5, the instrument had a 0.45 mm diameter, and at D16, it had a 0.9 mm diameter. D5 and D16 were only considered as the apical 5 mm was obturated and hence not included in the intracanal volume measurement. For the GET group, the EG6 had a head length of 2.5 mm, a tip diameter of 0.28 mm, and a back diameter of 0.71 mm. These diameters fall within the range of the D5 and D16 intervals (0.45-0.90 mm), but once the tip drill exceeds the D9 (0.61 mm) level in the apical direction, which is related to the third middle part of the canal, the back tip diameter of the EG6 (0.71 mm) starts to create additional lateral dentinal loss. This means that the intracanal volume of the GET was the result of the combination of the volume created by the final shaping 25/04 file in the coronal part and the volume created by the back tip diameter (0.71 mm) of the EG6 in the middle part of the canal. For the CET group, ED5 had a tip diameter of 0.40 mm and a back diameter of 1 mm, which are the same interval diameters as D5 and D16. However, because the ultrasonic tip was managed by a freehand operator, it could have produced lateral movements that further increased the dentinal loss. This, in turn, leads to a final diameter exceeding the insert’s tip, which means that the intracanal volume measured in the CET was also the resulting combination of the volume created by the final shaping file 25/04 and the volume created by the ED5 insert’s activity. In the present study, the shaping protocol affected the intracanal volume; however, it simulated the clinical scenario because every RCT required a shaping step.

For the same patient, the difference in intracanal volume between the GET and CET samples can be explained by the uncontrollable movement of the ultrasonic tip, which could create additional substance loss. The difference in the intracanal volume within the GET and CET samples varied despite the use of the same drill, ultrasonic tip, and final shaping file. This variation can be explained by the difference in tooth length between patients, the calcification level, and the variety of the initial intracanal anatomy before the RCT.

This study has several limitations. The small sample size, inherent to this in vivo pilot design, limited subgroup statistical analyses and the generalizability of the results. Only mandibular incisors with simple anatomy were included; therefore, the findings cannot be extrapolated to other tooth types. Dentinal substance loss was assessed using the indirect post-and-core technique, which may underestimate the true volume due to the inability to capture undercuts, but was chosen to comply with the ALARA principle. In addition, the recorded treatment duration for the GET did not include the time required for digital planning and guide fabrication. Finally, long-term clinical outcomes were not evaluated, highlighting the need for further studies with larger samples and extended follow-up.

## Conclusions

Within the limitations of this in vivo study, it is shown that GET has significantly demonstrated superior performance over CET in reducing substance loss and treatment duration compared to CET in calcified lower incisors of human teeth. Based on this small sample size, it may be optimal to use the CET when the calcification level encountered is in the first third of the middle part of the root canal because substance loss turned out to be negligible compared to the GET, and the overall treatment duration may be the same when the GET’s planning phase is factored in. For the second and third levels of the middle part, the GET is recommended because it is more conservative in terms of dentinal loss and more time-efficient. For the same calcification level in the third level of the middle part, GET exhibited a higher success rate than CET. Future studies with a larger sample size and diverse tooth morphologies are warranted to further validate these findings and could generate significant results concerning the GET’s applicability in various everyday endodontic challenges.
